# The social hierarchies and intra-group dynamics of symphony orchestras: a psychological perspective on ensemble cohesion

**DOI:** 10.3389/fpsyg.2026.1815293

**Published:** 2026-05-08

**Authors:** Ruiqi Pang, Qiuyan Wei

**Affiliations:** Saint Petersburg State Institute of Culture, Saint Petersburg, Russia

**Keywords:** ensemble cohesion, hierarchy, job demands–resources, social identity, symphony orchestra

## Abstract

Symphony orchestras require highly precise coordination under time pressure and can be analyzed through a high-reliability organizing lens, insofar as larger uncorrected disruptions in timing, balance, or entry may cascade into salient artistic breakdowns. Yet aural cohesion is produced within a stratified system involving conductors, principals, and section players, which can create subgroup boundaries and social fragmentation. This Mini Review integrates identity-based and job-demands–resources perspectives to explain how hierarchical control, evaluative exposure, perfectionistic norms, and role ambiguity increase burnout risk when autonomy, voice, fairness, and psychological safety are constrained. We propose the Orchestral Dynamics Moderation Model, in which shared leadership and high social identity clarity buffer the pathway from hierarchical stressors to burnout and performance variability, and we identify bullying and mobbing as an under measured structural risk. We conclude by outlining priorities for longitudinal, multimethod, and cross-cultural research, including naturalistic psychophysiology such as HRV and cortisol and intervention trials.

## Introduction

### The orchestra through a high-reliability lens

Symphony orchestras perform under live conditions characterized by tight coupling among parts, strong temporal interdependence, and high demands for coordination precision. In rehearsal and performance alike, musicians must anticipate, monitor, and rapidly adjust to one another under conditions of limited recovery time and public evaluative exposure. For this reason, ideas from the high-reliability organization literature can serve as a useful analytic lens for understanding orchestral coordination as a form of ongoing error monitoring and correction rather than as purely aesthetic collaboration (Dwyer et al., 2023; Fricke et al., 2025).

At the same time, this comparison should be treated as an analytic analogy rather than a literal organizational classification. Classical high-reliability organizations typically operate in settings where failures may have catastrophic safety consequences, whereas orchestral disruptions are more appropriately understood as artistic, reputational, or coordination-related failures. The value of the analogy therefore lies not in equating orchestras with safety-critical systems, but in highlighting their demanding combination of tight temporal synchrony, distributed interdependence, rapid error detection, and limited opportunity for recovery in live performance.

Importantly, not all timing deviations should be treated as failures within this analogy. Professional ensemble performance is not perfectly isochronous. Studies of ensemble coordination show that small between-player asynchronies are common even in expert settings, and that performers maintain togetherness through continuous micro-adjustments rather than exact simultaneity at every event (Repp and Keller, 2008; Timmers et al., 2014; Wing et al., 2014). Such micro-asynchronies may reflect expressive timing, perceptual alignment, or leader-follower dynamics rather than breakdown *per se*. Accordingly, in the present review, “failure” refers more narrowly to larger, insufficiently corrected coordination disruptions that become musically salient, not to ordinary expressive temporal variability.

Used in this bounded sense, the high-reliability lens helps clarify why orchestral settings can generate both exceptional coordination and considerable psychological strain. The same performance ecology that requires rapid adaptation, vigilance, and precision may also intensify evaluative pressure, constrain opportunities for voice, and heighten sensitivity to hierarchical control. This makes the HRO-informed perspective conceptually useful for analyzing how coordination demands intersect with leadership structure, subgroup dynamics, and occupational wellbeing in symphony orchestras.

### The paradox of cohesion

Orchestras pursue a high degree of aural cohesion, but their stratified structure, including seating hierarchies and uneven allocation of section resources, can foster social fragmentation. Evidence suggests that when members perceive the ensemble as divided into subgroups, overall team identification declines and emotional exhaustion increases (Tiede et al., 2021). For professional classical musicians, such organizational and relational demands can become important sources of stress, although the available evidence remains context-specific. For example, qualitative evidence from professional classical musicians highlights organizational, performance, and relationship demands, but this evidence should not be conflated with findings from educational orchestra settings (Willis et al., 2024). Related work in music education further suggests that seating arrangements can shape perceptions of visibility, marginality, and participation, although that evidence comes from a United States public-school string orchestra program rather than professional symphony orchestras (Yi, 2023).

### Objectives and framework

This Mini Review examines orchestral hierarchy and ensemble cohesion through a staged theoretical framework. First, Social Identity Theory and Self-Categorization Theory are used to explain how sectional identities form, why musicians may anchor belonging at the local section level, and how subgroup boundaries can either support coordination or fragment orchestra-wide cohesion (Tajfel and Turner, 1979; Turner et al., 1987). Second, Realistic Conflict Theory is introduced to explain how these subgroup distinctions may become competitive when sections are perceived to vie for scarce resources, such as conductor attention, symbolic prestige, visibility, or influence over artistic decisions. Third, the Job Demands-Resources model is used to analyse how hierarchical pressure, evaluative exposure, role ambiguity, constrained autonomy, and limited voice may translate these structural and intergroup tensions into psychological strain, emotional exhaustion, and burnout (Bakker et al., 2023; Demerouti and Bakker, 2023; Bakker and Demerouti, 2024). Finally, the proposed Orchestral Dynamics Moderation Model integrates these perspectives by specifying how hierarchical centralization may heighten identity threat and occupational strain, and how shared leadership and high social identity clarity may buffer these pathways. In this way, the review moves from subgroup formation, to intergroup competition, to strain processes, and then to an integrative orchestral-specific conceptual model.

## Typology of orchestral hierarchy, power, and leadership

Orchestral hierarchy can be conceptualized as a coupled system of power and control that operates at multiple levels. Conductors shape macro level interpretation and overall coordination. Principals provide meso level leadership that aligns sectional work with the broader artistic plan. Section players implement micro level execution under strong temporal and coordination constraints.

### The conductor: transformational and transactional leadership

Within the conductor musician relationship, a practical typology contrasts transformational leadership, which emphasizes vision and meaning making, with transactional leadership, which emphasizes error correction and rule enforcement. Evidence suggests that transformational leadership does not uniformly translate into higher artistic outcomes. In a study of 22 German symphony orchestras, transformational leadership interacted with musicians positive group affect. Higher external ratings of artistic quality emerged primarily when both transformational leadership and positive group affect were high, whereas increasing either factor alone did not yield comparable gains (Boerner and von Streit, 2007).

A complementary pathway emphasizes directive and authoritative coordination. In a study of 30 German orchestras, directive charismatic leadership was positively associated with evaluations of performance quality and artistic quality, which suggests that centralized coordination may reduce coordination costs in highly synchronized and time constrained task systems (Boerner et al., 2004).

More recent evidence has focused on musician preferences and perceived acceptability. A mixed methods study with South African professional musicians using the MLQ and interviews found that musicians tended to value idealized influence and inspirational motivation. They highlighted the importance of trust, adequate preparation, and flexible adjustment to rehearsal conditions, while also noting that conductors must manage a narrow boundary between excessive interference and excessive permissiveness (Barrett-Berg and van der Merwe, 2023). Claims about how conductor charisma and authority influence cohesion through fear or admiration are still grounded mainly in interviews and normative accounts, such as ethics centered leadership proposals, rather than in causal evidence based on physiological indicators such as cortisol or HRV (de Caro and Palazzolo, 2024). This limitation aligns with broader research on professional musicians' mental health, where self-report measures dominate and authors call for more objective behavioral and physiological assessment. Taken together, these findings suggest that conductor leadership matters for ensemble functioning, but the evidence base remains context-bound, drawing mainly on German orchestra survey studies and South African mixed-methods data rather than on cross-national comparative research.

### The principal: the linking pin role

The principal role is defined less by superior individual performance and more by its structural position as a linking function. Upward, principals translate the conductor overall vision into actionable technical rules and stylistic boundaries for the section. Downward, they aggregate section input, manage interpersonal friction, and implement real time correction during rehearsal.

Drawing on the classic “linking pin” conception of managers as actors who connect adjacent organizational levels, as well as later work on middle managers' boundary-spanning, integrative, and interpretive roles, the principal can be understood as occupying a structurally hybrid position between conductor and section (Likert, 1961, 1967). In the present review, we use two heuristic labels—Translator-Coach and Gatekeeper-Enforcer—to capture contrasting emphases within this linking function, rather than to propose a formal typology. The Translator-Coach emphasis highlights explanation, demonstration, feedback, and psychological safety in reducing uncertainty within the section, whereas the Gatekeeper-Enforcer emphasis highlights discipline, standardization, and boundary control in the service of consistency.

This role is also characterized by dual loyalty and role conflict, because it requires commitment to the conductor goals while maintaining relational obligations to section members. These demands can produce a resource depletion pattern in which pressure flows downward while resistance and expectations flow upward. Direct quantitative research on principals remains limited, but evidence from broader middle management research is informative. Longitudinal data indicate that role overload increases workplace anxiety and is associated with resistance to organizational change (Wang et al., 2024). If comparable mechanisms operate in orchestral settings, principals may be particularly vulnerable to anxiety and emotional labor during high standard, high surveillance, low tolerance rehearsal cycles. This remains a theoretically plausible inference rather than a conclusion established by direct orchestral evidence. If so, this could weaken their capacity to function as identity representatives and cohesion builders within the section, although this inference requires direct testing in orchestral samples.

### The section player autonomy suppression

Section players face a high skill low control condition. Their training is highly specialized, yet they have limited control over interpretive decisions, rehearsal pace, and acceptable error margins, especially when rehearsals are strongly centralized. From a work design perspective grounded in the Job Demands Resources and job characteristics traditions, autonomy and developmental opportunities function as core resources. When these resources remain chronically low while demands stay high, the risk context for burnout and anxiety becomes more likely.

Evidence for reduced autonomy in orchestral settings should be interpreted in a context-specific way. In a cross-sectional study of Danish professional symphony orchestra musicians, participants reported higher emotional demands and lower influence, social support, and job satisfaction than the Danish general workforce (Holst et al., 2012). Within that Danish sample, instrument-group differences also emerged: second violinists showed relatively lower scores on influence at work and possibilities for development than several other instrument groups, suggesting that not all orchestral positions are experienced equally in terms of job control and developmental opportunity (Holst et al., 2012). Complementing this, a large Norwegian study found that orchestral players reported less control than other musician roles within the musician sample, again pointing to autonomy constraints in large ensemble settings (Détári et al., 2020). Taken together, these findings support a cautious structural account in which reduced control and uneven developmental opportunities may be salient in some professional orchestral contexts, although such patterns should not be generalized across national systems without further comparative evidence.

## Intra-group dynamics: social identity and sectional subcultures

### The section as a psychological home in group identification formation

From the perspectives of Social Identity Theory and Self Categorization Theory, orchestras are composite systems that contain multiple cohesive subgroups. Because interaction density is high and functional interdependence is strong, sections can become a psychological home for musicians. Under high pressure, individuals often anchor their self-definition at this local level, which yields a pattern in which section identification precedes orchestra wide identification (Tajfel and Turner, 1979; Turner et al., 1987).

Organizational psychology research indicates that perceived subgroup divisions can indirectly increase emotional exhaustion by reducing overall team identification (Tiede et al., 2021) or by increasing task conflict (Schulte et al., 2020). This implies that when sectional identification is strong while orchestra wide identification is weakened, a division of labor that is designed to support precise coordination can become a pathway to psychological depletion.

At the level of sectional subcultures, early claims about stable personality differences across instrument groups (Kemp, 1981) appear to be amplified by group stereotypes (Butković and Modrušan, 2019). More recent research emphasizes broader trait profiles among professional musicians and their links to performance anxiety (Spahn et al., 2024). For orchestral dynamics, sectional identity and instrument related stereotypes provide rapid cues for self-categorization. These cues shape how musicians interpret cross sectional collaboration and can escalate technical disagreements into perceived identity invalidation. When such subgroup identities become linked to unequal access to attention, recognition, visibility, or influence, the issue is no longer only one of self-categorization and belonging, but also one of intergroup competition over scarce resources. This provides the rationale for turning to Realistic Conflict Theory in the following section.

### Inter group competition and stereotyping from realistic conflict theory to conflict typology

Building on the identity processes outlined above, Realistic Conflict Theory helps explain when sectional differentiation may shift from benign functional specialization to antagonistic intergroup competition. In orchestral settings, this may occur when sections perceive themselves as competing for scarce organizational or symbolic resources, including conductor attention, high-visibility passages, prestige, status, or influence over rehearsal priorities and interpretive decisions (Sherif et al., 1961). More recent work continues to support the broader proposition that perceived competition over scarce resources can intensify intergroup opposition, although such evidence comes from non-orchestral settings (e.g., Hendriks et al., 2024).

Conflict typologies distinguish task conflict from relationship conflict (Jehn, 1995; De Dreu and Weingart, 2003). Task conflict about performance plans can, in principle, support artistic alignment, but meta-analytic evidence indicates that it is often associated with lower satisfaction. When technical disagreements become moralized, they can escalate into relationship conflict that undermines psychological safety. These forms of conflict often co-occur, because technical disputes that align with sectional boundaries can be interpreted as out group challenge. The pathway from subgroup perception to task conflict to emotional exhaustion (Schulte et al., 2020) captures how rehearsal disagreements can develop into sustained psychological strain.

Although much work emphasizes the negative consequences of conflict, recent meta-analytic evidence indicates substantial situational heterogeneity in conflict effects (Yuan et al., 2026). Future research should therefore operationalize healthy competition in orchestras with explicit boundary conditions that include low identity threat, high psychological safety, and clear superordinate goals. It should then test these conditions across repertoires and orchestras using behavioral coding, social network analysis, and physiological stress indicators.

## Structural stressors, burnout and performance

### From hierarchy to burnout: JD-R model's mapping to orchestras

In the Job Demands Resources framework, occupational burnout is not simply a function of technical difficulty. It more often reflects sustained demands combined with insufficient resources (Bakker et al., 2023). In symphony orchestras, hierarchical structure can be operationalized as a set of visible and recurring organizational demands. These demands include perfectionistic pressure under stringent expectations for coordination precision, intensive error correction under limited rehearsal time, continuous evaluative exposure to conductors and principals, and the emotional labor and sustained vigilance required in performance settings. Here, coordination precision should not be equated with literal simultaneity, because professional ensemble timing normally involves adaptive micro-variability and recurrent correction processes even when performance is perceived as highly together (Timmers et al., 2014; Wing et al., 2014). At the same time, hierarchy can systematically constrain core resources. These resources include decision latitude and control, influence over interpretive plans, opportunities for recognition and social support, and cross-sectional trust and collaborative capital.

Although most quantitative studies in orchestral contexts remain cross-sectional and correlational, available Scandinavian evidence points in a similar direction. In Norway, a large epidemiological study comparing 1,607 members of the Norwegian Musicians' Union with the general workforce found that musicians perceived their work as more demanding and less well supported, and that orchestral players reported less control than some other musician roles (Détári et al., 2020). In Denmark, cross-sectional survey research with professional symphony orchestra musicians likewise found that emotional demands were associated with subjective stress, while workplace social capital buffered this association (Pihl-Thingvad et al., 2022). These findings support a context-bounded structural account in which burnout risk in some professional orchestral systems appears to be shaped less by musical difficulty *per se* than by the combination of high demands, constrained control, and limited social resources.

These studies remain correlational, so they can establish associations but not a causal pathway from hierarchical structure to burnout. Causal tests will require longitudinal tracking, natural experiments such as conductor turnover or organizational reform, and multilevel models that separate effects at the individual, section, and orchestra levels.

### Psychological safety and performance quality from dare to speak to can learn

Psychological safety can be treated as a core social resource in orchestras. When members believe that raising concerns, acknowledging mistakes, and offering dissent will not trigger humiliation or punishment, teams are more likely to exchange high quality information and correct errors quickly. Organizational research suggests that such conditions can support learning, information exchange, and adaptive performance. In strongly hierarchical artistic organizations, psychological safety can be undermined by a pattern in which accountability flows downward while silence flows upward. Section players may withhold upward voice because they anticipate negative evaluation. Principals may also filter or avoid conflict laden information because they face dual loyalty demands. These processes can slow learning and reduce the rate at which problems are surfaced during rehearsal.

Evidence from outside orchestral settings suggests a robust negative association between psychological safety and burnout-related outcomes, but this literature is not orchestra-specific. For example, American Psychological Association's (2024) Work in America survey concerns United States workers broadly, de Lisser et al. (2024) examined clinicians in healthcare settings, and Bahadurzada and Edmondson (2024) drew on longitudinal employee data under resource constraints. Accordingly, the present manuscript uses this literature as converging cross-industry evidence that psychological safety may matter in orchestras, while recognizing that direct longitudinal tests in professional symphony orchestras remain scarce.

### Silent variables internal bullying and mobbing in orchestras

When hierarchical pressure persists and voice channels are weak, strain may be displaced laterally within the group. This can appear as exclusion, humiliation, chronic belittlement, or coordinated mobbing and bullying. Such behaviors are often difficult to detect. They may not be formalized in organizational rules, yet they can meaningfully alter perceptions of safety, dignity, and belonging. They can also contribute to a risk pathway that includes exhaustion, depressive symptoms, and intentions to exit.

In a Finnish cross-sectional comparison, musicians from all domestic Finnish symphony orchestras reported a higher likelihood of psychological violence or bullying than the Finnish general workforce (OR = 3.0, 95% CI [2.0, 4.7]; Vastamäki et al., 2023). Because this study was cross-sectional and embedded in the Finnish orchestral labor context, it does not justify a universal claim that hierarchy causes bullying across orchestra systems. It does, however, indicate that bullying should be treated as a potentially structural organizational risk rather than only as an individual personality issue.

## Discussion: synthesis and theoretical model

### Synthesis

Current evidence suggests that hierarchical differentiation in symphony orchestras is not only a structural condition that enables complex coordination. It can also accumulate psychological costs through an imbalance between job demands and resources. Stringent expectations for precision, continuous evaluative exposure, and unclear role boundaries can increase stress. Importantly, such expectations concern the rapid management of musically consequential coordination problems, not the elimination of all micro-asynchrony, because expert ensemble performance normally relies on ongoing adaptive timing adjustments (Repp and Keller, 2008; Wing et al., 2014). Limited opportunities to speak up and restricted autonomy can weaken resource replenishment, which can promote emotional exhaustion and intentions to leave. Importantly, evidence from Finnish domestic symphony orchestras indicates a higher prevalence of psychological violence and bullying than in the Finnish general workforce, suggesting that hierarchical pressure may, in at least some orchestra systems, spill over into lateral aggression and further undermine trust and cohesion (Vastamäki et al., 2023). Social Identity Theory further indicates that musicians often experience stronger identity security and belonging at the level of sections and small groups. When orchestra wide identity boundaries and norms are unclear, identity uncertainty can amplify in group and out group distinctions and stereotyping. This can intensify the tension between aural cohesion and social fragmentation (Hogg, 2023).

### Proposed conceptual model orchestral dynamics moderation model

The Orchestral Dynamics Moderation Model is proposed here not as a replacement for the preceding theories, but as an integrative framework tailored to orchestral settings. It extends Social Identity Theory and Self-Categorization Theory by focusing not only on how sectional identities form, but also on how low orchestra-wide identity clarity and heightened identity threat may emerge under hierarchical centralization. It incorporates Realistic Conflict Theory as a conditional mechanism that becomes especially relevant when sections perceive themselves as competing for scarce symbolic or organizational resources. At the same time, it reinterprets the orchestral context through a Job Demands-Resources lens by specifying how centralized control, evaluative exposure, role ambiguity, and restricted voice function as demands, whereas shared leadership, psychological safety, and social identity clarity function as buffering resources. In this sense, the model does not simply add another theoretical layer; rather, it synthesizes identity, competition, and strain mechanisms into a single orchestral-specific account of how hierarchy may affect burnout risk and performance variability.

Two moderators are expected to weaken this pathway. Shared leadership distributes influence over rehearsal decisions and problem solving across members. This can buffer increases in exhaustion when workload rises. High social identity clarity makes the collective identity and shared standards explicit through identity-based leadership and identity construction. This strengthens team identification and is associated with lower burnout.

## Future research agenda

Future work should advance on three fronts, including methodology, cross cultural scope, and intervention design. Methodologically, research should move beyond self-report cross sectional designs and adopt multimethod, contextual, and multilevel approaches. Studies can combine experience sampling, behavioral performance measures, and acoustic indicators during rehearsal and performance, and they can incorporate wearable or field-based psychophysiology such as HRV and salivary cortisol to capture real time stress responses and coordination dynamics (Sebastiani et al., 2022; Walther et al., 2024). Existing work has already demonstrated the feasibility of such designs in non-professional orchestra concert settings (Walther et al., 2024), but comparable studies in professional symphony orchestras remain needed.

Theoretically, samples and contexts should be expanded beyond WEIRD settings (Henrich et al., 2010). Cross cultural comparisons can test how orchestral institutions such as unionization and contract stability and cultural norms such as collectivism reshape acceptance of hierarchy, strength of sectional identification, and conflict expression. These tests can clarify the boundary conditions of the Orchestral Dynamics Moderation Model.

Finally, the field should shift from mechanism focused work to testable intervention programs. Cluster randomized and stepped wedge designs are well suited for evaluating identity leadership-based team clarification, shared leadership reflection practices, anti-bullying and bystander intervention training in professional orchestras (Nielsen et al., 2024). Interventions used in music performance anxiety research, including mindfulness-based programs, should also be evaluated for effectiveness in orchestral settings (Kinney et al., 2025; Stanson et al., 2022).
